# Molecular diagnosis of central nervous system opportunistic infections and mortality in HIV-infected adults in Central China

**DOI:** 10.1186/s12981-017-0150-2

**Published:** 2017-05-02

**Authors:** Rongrong Yang, Hong Zhang, Yong Xiong, Xien Gui, Yongxi Zhang, Liping Deng, Shicheng Gao, Mingqi Luo, Wei Hou, Deyin Guo

**Affiliations:** 1 Department of Infectious Diseases, Zhongnan Hospital of Wuhan University, 169 Donghu Road, Wuhan, 430071 China; 2 Basic Medical College of Wuhan University, Wuhan, China

**Keywords:** AIDS, Central nervous system diseases, Cerebrospinal fluid

## Abstract

**Background:**

CSF PCR is the standard diagnostic technique used in resource-rich settings to detect pathogens of the CNS infection. However, it is not currently used for routine CSF testing in China. Knowledge of CNS opportunistic infections among people living with HIV in China is limited.

**Methods:**

Intensive cerebrospiral fluid (CSF) testing was performed to evaluate for bacterial, viral and fungal etiologies. Pathogen-specific primers were used to detect DNA from cytomegalovirus (CMV), herpes simplex virus (HSV), varicella-zoster virus (VZV), Epstein-Barr virus (EBV), human herpesvirus 6 (HHV-6) and John Cunningham virus (JCV) via real-time polymerase chain reaction (PCR).

**Results:**

Cryptococcal meningitis accounted for 63.0% (34 of 54) of all causes of meningitis, 13.0% (7/54) for TB, 9.3% (5/54) for Toxoplasma gondii. Of 54 samples sent for viral PCR, 31.5% (17/54) were positive, 12 (22.2%) for CMV, 2 (3.7%) for VZV, 1 (1.9%) for EBV, 1 (1.9%) for HHV-6 and 1 (1.9%) for JCV. No patient was positive for HSV. Pathogen-based treatment and high GCS score tended to have a lower mortality rate, whereas patients with multiple pathogens infection, seizures or intracranial hypertension showed higher odds of death.

**Conclusion:**

CNS OIs are frequent and multiple pathogens often coexist in CSF. Cryptococcal meningitis is the most prevalent CNS disorders among AIDS. The utility of molecular diagnostics for pathogen identification combined with the knowledge provided by the investigation may improve the diagnosis of AIDS related OIs in resource-limited developing countries, but the cost-efficacy remains to be further evaluated.

## Background

Central nervous system (CNS) infectious is a major cause of morbidity and mortality among people living with human immunodeficiency virus (HIV) [1, 2]. Symptomatic neurological diseases happened in about 40–70% of HIV/AIDS patients during the course of their illness [1], and about 10–20% HIV/AIDS patients have neurologic symptoms as an initial manifestation [2, 3]. Studies indicated that opportunistic infections are the most common causes of neurologic diseases in HIV/AIDS patients in developing countries [4–8]. In China, lumbar puncture (LP) is a routine practice and cerebrospinal fluid (CSF) is commonly collected for patients with suspected diagnosis of CNS infection. However, the available and frequently-used means of diagnosis are generally limited to CSF routine and biochemical tests, Gram stain, India ink stain, cryptococcal antigen (CrAg) detection, and bacterial/fungal cultures. As a result, country-specific data in our hospital regarding prevalence of CNS disorders are sparse, and many patients remain misdiagnosed and inadequately treated.

In resource-rich settings, the standard diagnostic technique used to identify the presence of viral, parasitic, and specific bacterial infection of the CNS disorders is CSF polymerase chain reaction (PCR); however, in China in HIV-infected patients, CSF PCR is not currently used for routine tests. The prevalence data regarding CNS infectious diagnosed via PCR in China was particularly absent at present. In this study, CSF samples were tested for CMV, HSV, VZV, EBV, HHV-6 and JCV via PCR. The prevalence of CNS OIs in HIV-infected Chinese adults and their impact on patient outcomes were determined.

## Methods

### Patients population

This hospital-based study was conducted at Zhongnan Hospital of Wuhan University, China, between 1 December 2012 and 30 September 2014. Patients were eligible for the study if they had: (1) confirmed with HIV infection; (2) three or more symptoms of meningitis and/or encephalitis (headache, seizure, nausea/vomiting, alteration in consciousness, photophobia, or a focal neurological deficit); and (3) agreed to do a lumbar puncture(LP), as determined by the attending physician. Exclusion criteria for enrollment included: (1) patients with delirium, such as sepsis due to a non-neurological infection or metabolic abnormality; (2) patients with peripheral neuropathy and psychosis rather than medical explanation for their symptoms; or (3) patients with a documented immunosuppressive except for HIV infection or neurosurgical illness. Meningitis was diagnosed if there was a combination of meningeal irritation symptoms and signs and the presence of more than five white blood cells in CSF obtained by lumbar puncture. Exclusion criteria included malignancies, various inflammatory conditions, sarcoidosis, and infectious secondary to procedures. Meningoencephalitis was defined as any brain parenchymal involvement (e.g. seizures), behavioral change, confusion, dysphasia and other cortical deficits, with signs of meningeal irritation. Encephalitis was defined as fever with altered sensorium with CSF pleocytosis and raised protein with or without focal neurological signs in whom malaria, bacterial or fungal meningitis were excluded. Trained radiologists and neurologists were also invited to help identifying patients with a suspected diagnosis of meningitis, encephalitis or meningoencephalitis. The study was approved by Zhongnan Hospital of Wuhan University Ethics Committee.

Demographic information, such as age, gender, marital status, risk factors, duration between HIV infection and hospital admission, clinical features and past medical history were collected on a standardized report form by the trained physician. In China, lumbar puncture (LP) is routine practice for patients with a suspected CNS infection prior to antibiotic treatment. Other important medical assessment were computed tomography (CT) or magnetic resonance imaging (MRI) for patients with a focal brain lesion or suspected brain hemorrhage. But whether the imaging examination ultimately used depends on the patients’ or their family members’ willingness and attitude.

### CSF testing

All CSF samples were collected via lumbar puncture and stored at −20 °C for molecular diagnostics. DNA was isolated by using the easyMAG Instrument (bioMérieux). Primers amplifying a conserved sequence of viral DNA polymerase (BALF5) gene and a fluorogenic probe were used at a primer concentration of 0.9 μM and a probe concentration of 0.25 μM. Amplification was carried out in an ABI PRISM 7900 HT Sequence Detector (PE Applied Biosystems) and the standard curve was created with Sequence Detection System software by plotting the CT values against known CMV, EBV, JCV, VZV, HHV-6, HSV or TB-DNA concentrations. The assay has a detection limit of 500 genome equivalents/ml.

### Statistical analysis

Continuous data are presented as mean ± standard deviation (for normally distributed variables) or median and interquartile range or IQR (for variable influenced by extreme values). Categorical data are presented as numbers with proportions. *P*-values were 2-sided and considered statistically significant if <0.05. Statistical analyses were performed using SPSS for Windows, version 19.0 (SPSS, Chicago, IL).

## Results

### Patient characteristics

**Fig. 1:**
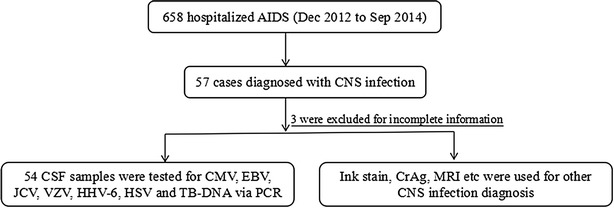
Study profile

In this study, 57 patients with CNS diseases out of 658 hospitalized AIDS (≥14 years old) were admitted to Zhongnan Hospital of Wuhan University with a complaint of new or recurrent neurological or psychiatric symptoms were included (Fig. 1). Of these 57 patients, complete clinical information was available in 54 (94.7%). Forty-one cases of meningitis, six cases of encephalitis and seven cases of meningoencephalitis were found in this series. Characteristics of the 54 patients are shown in Table 1. The mean age was 38 years and 64.8% of participants were male. The main occupational population was farmer (70.4%). And the main HIV acquisition route was sexual contact (79.6%). At the time of enrollment, a median duration of 45 days was observed. The median CD4^+^ T cell count was 31 cells/μl.

**Table 1:** Patient demographics (N = 54)

Feature	No (%)
Age (years), mean (SD)	38 (11.2)
Male	35 (64.8)
Occupation	
Farmers	38 (70.4)
Clerk	8 (14.8)
Businessman	4 (7.4)
Others	4 (7.4)
HIV transmission route	
Sex	43 (79.6)
Blood donation/transfusion	6 (11.1)
IDU	2 (3.7)
Unknown	3 (5.6)
Days since HIV diagnosis, median (IQR)	45 (1–732)
CD4^+^ T cells/μl, median (IQR)	31 (21–83)

*SD* standard deviation

### Pathogen(s) detecting in CSF

Pathogens were detected in 47 (87.0%) CSF samples. The number of patients diagnosed with one or more pathogens is shown in Table 2. *Cryptococcus neoformans* was the most common pathogen found (63.0%) and the most common copathogen (22.2%). There were high rates of detection of CMV (22.2%) and tuberculosis (13.0%). We also detected VZV (3.7%), JCV (1.9%), EBV (1.9%) and HHV-6 (1.9%) in this patient population. No case of HSV were detected by PCR.

**Table 2:** Characteristics of patients with CSF pathogen(s)

Pathogens	Individual CSF prevalence	1 pathogen	No. of cases	2 pathogens	No. of cases	3 pathogens	No. of cases
Crypto^a^	34/54 (63.0%)	Crypto	22	Crypto/CMV	7	Crypto/TB/CMV	2
				Crypto/VZV	1	Crypto/TB/HHV-6	1
				Crypto/JCV	1		
CMV	12/54 (22.2%)	CMV	2	CMV/TB	1		
TB	7/54 (13.0%)	TB	3				
Toxop	5/54 (9.3%)	Toxop	5				
VZV	2/54 (3.7%)	VZV	1				
JCV	1/54 (1.9%)	JCV	0				
EBV	1/54 (1.9%)	EBV	1				
HHV-6	1/54 (1.9%)	HHV-6	0				
HSV	0/54 (0%)	HSV	0				
Total	63 pathogens	34/54 (63.0%)		10/54 (18.5%)		3/54 (5.6%)	

### Results of PCR positive pathogen(s) detected by other methods

Of the 34 cases with cryptococcus, three were negative on India Ink (8.8%), and four were negative on CrAg (11.8%).

Of five PCR diagnosed toxoplasmosis in this series, all responded clinically and radiographically within four weeks of initiation of pyrimethamine and clindamycin combination therapy.

Among the 12 CMV-DNA positive patients, 8 (66.7%) serum samples were negative for CMV-DNA and the average level of CMV-DNA in CSF was 2.80 ± 1.15 Log_10_copies/ml.

Among five patients diagnosed toxoplasmosis, brain CT or MRI was used for four patients. The representative ring enhancing lesions were found in all the four patients.

For the EBV-DNA positive patient, mass lesions were not found. For the JCV-DNA positive patient, typical white matter changes were found, which was consistent with the PCR for JCV.

Among 12 CMV-DNA positive patients, brain CT or MRI was used for 6 patients and none was found with brain parenchymal lesion.

### Risk factors associated with mortality

In our study population, the total mortality rate was 22.2% (12/54). Table 3 summarizes the potential risk factors of death in our study population. The odds of death together with 95% confidence intervals were reported for each covariate. In the multivariate analysis, pathogen-based treatment and high glasgow coma scale (GCS) score were associated with lower odds of death. Conversely, patients with multiple pathogens infection, seizures or intracranial hypertension showed higher odds of death.

**Table 3:** Variables associate with death in univariate and multivariate analysis

Variable	Univariate analysis		Multivariate analysis	
	Odds ratio (95% CI)	*P* value	Odds ratio (95% CI)	*P* value
Categorical				
Male	0.58 (0.30–1.32)	0.26		
cART status	0.48 (0.30–2.22)	0.56		
Multiple pathogens	0.33 (0.14–0.56)	<0.0001	0.46 (0.26–0.74)	0.002
Cryptococcal meningitis	1.32 (0.80–2.34)	0.22		
CMV	1.21 (0.58–2.33)	0.74		
Tuberculosis	0.63 (0.88–3.26)	0.30		
EBV	0.70 (0.44–1.33)	0.23		
Other pathogens	0.85 (0.44–1.63)	0.53		
Pathogen-based treatment	5.7 (2.0–17.6)	<0.0001	4.8 (2.4–16.5)	0.001
Seizures	0.87 (0.33–1.42)	0.08	0.52 (0.36–0.98)	0.04
Intracranial hypertension	0.27 (0.18–0.84)	0.04	0.33 (0.22–0.84)	0.03
Disturbance of consciousness	0.67 (0.43–1.04)	0.06	1.00 (0.46–2.18)	0.99
Continuous				
Age	0.36 (0.14–0.89)	0.08	0.56 (0.32–1.02)	0.25
GCS	0.22 (0.12–0.32)	<0.0001	0.42 (0.21–0.80)	0.001
CD4 count	0.66 (0.45–1.24)	0.68		

*GCS* Glasgow coma scale

## Discussion

This hospital-based study provides information about the spectrum of pathogens causing CNS infections and mortality among people living with HIV in Zhongnan Hospital of Wuhan University in central China. The spectrum of HIV-associated central nervous system disorders may be influenced by differences in the epidemiology.

We identified a microbiologic diagnosis for clinical meningitis in a cohort of HIV-infected Chinese inpatients. Our data suggested that the most common pathogen causing meningitis is Cryptococcus neoformans. The most common viral pathogen causing meningitis is CMV and that the most common bacterial pathogen is TB. Our finding highlighted the utility of molecular diagnostics for improving pathogen identification for CNS infections.

The HIV epidemic has caused a marked increase in cases of cryptococcal meningitis such that it has become the leading cause of meningitis among patients living with HIV [9–11]. We found that cryptococcal meningitis caused the majority of meningitis among hospitalized AIDS patients, which accounted for 63.0% of all causes of meningitis in this study. In a review, the total cryptococcal meningitis prevalence was 52.0% in sub-Saharan Africa [12]. Our relatively high prevalence may be attributable to different methodologies and tests used for assessing prevalence; differences in host factors; or geographic variations in the epidemiology of cryptococcal meningitis.

The prevalence of toxoplasma gondii in this study are higher than previously reported from a study in Zambian [13]. In our study population, the median CD4^+^ T-cell count was 31 cells/μl and all were newly diagnosed with HIV and without any prophylaxis treatment, whereas in Zambian [13] study, the enrollment CD4^+^ T-cell count was 81 cells/μl in men and 99 cells/μl in women, and 118 (35.6%) patients were on cART for a median duration of 240 days. The patient population and the presence of anti-Toxoplasma treatment may account for the high present of *Toxoplasma gondii*.

Cytomegalovirus is a ubiquitous agent that can cause infection at any time during the course of life and commonly infects individuals from diverse geographical and socio-economic backgrounds [14, 15]. By serology, a study reports that 93.9% of the HIV infection patients were positive for anti-CMV IgG antibody [16]. But in most patients, the diseases caused by CMV is so mild that it is overlooked. AIDS patients are commonly positive for CMV when sick with other illnesses. Diagnosis of CMV infection from tissue biopsies is considered the gold standard with specificity for histopathological evaluation near 100% but with low sensitivity (23.2%) [17]. In this study through PCR for CMV-DNA, 12 out of 54 (22.2%) CSF samples were positive. Whether the CMV is truly pathogenic or simply an innocent bystander remains to be further identified. Among the 12 patients with CMV-DNA positive in CSF, 8 (66.7%) serum samples were negative for CMV-DNA and the average level of CMV-DNA in CSF was 2.80 ± 1.15 Log_10_ copies/ml. Among 12 CMV-DNA positive patients, brain CT or MRI was used for six patients. None patient was found with brain parenchymal lesion. These features supported a low CMV burden which is combination with the negative PCR in the blood favored the argument of CMV being a bystander.

Cerebral toxoplasmosis is a very relevant neurological disease in individuals with AIDS [18]. In this study, toxoplasma is the fourth most common pathogen in CNS disorders. Among five patients diagnosed toxoplasmosis, brain CT or MRI was used for four patients. The representative ring enhancing lesions were found in all the four patients. The limited data imply PCR for the diagnosis of toxoplasmosis was accurate and reliable. Of 5 PCR diagnosed toxoplasmosis in this series, all responded clinically and radiographically within four weeks of initiation of pyrimethamine and clindamycin combination therapy. Although DNA positive in serum or cerebrospinal fluid can be diagnosed as Toxoplasma infection, the policy of empiric treatment of suspected Toxoplasma encephalitis is still satisfactory and recommendable. For patients with AIDS and suspected Toxoplasma encephalitis, early diagnosis and initiation of empiric treatment and antiretroviral therapy are important for good prognosis.

EBV is consider to be associated with primary CNS lymphoma in HIV infected patient, though further diagnostics for evaluation are lacking in this study. Detection of EBV DNA from a CSF sample may represent replication of EBV within B lymphocytes, and it was also closely related to an increased risk of death [19]. Neuroimaging was not obtained as part of this study. However, in the EBV PCR-positive patients with neuroimaging, mass lesions were not found. Therefore, we speculated that EBV might be a non-pathogenic marker of immunodepression, being shed from activated B-cells trafficking into the CSF during meningitis. Further investigation on the significance of EBV in the CSF is needed. Quantitative and comparison values from the CSF and serum is necessary to distinguish those with CNS lymphoma from those with non-pathogenic presence of EBV.

HIV strain and host susceptibility factors were thought to be less tendentious to infection by JCV, the pathogen of progressive multifocal leukoencephalopathy (PML). Indeed, the JCV CSF-positive patient had neurological symptoms consistent with PML. The sole JCV-DNA positive patients received brain imaging examination, white matter changes were found, which was consistent with the PCR for JCV.

Seroprevalence of CNS HSV infection has previously been documented at 47.2% in an urban Zambian population [20]. However, HSV DNA was not detected in any CSF samples in our study population. This is likely because the sample in our series is small and because our study used a different detection method (HSV-DNA PCR in CSF) as the Zambian population [20] (HSV IgG ELISA in serum).

In our study population, the total mortality rate was 22.2% (12/54). This rate of mortality is lower than reported in Zambian at the beginning of the AIDS epidemic [13]. Our study was not designed for long-term follow-up. It is possible that death occurred in the post-hospitalization period due to loss of follow-up, poor compliance with treatment, and poor access to emergency health services. As expected, the actual mortality was higher than 22.2%.

Our study shows that numerous pathogens can be found in the CSF of HIV-infected patients presenting with neurological symptoms in China, and the mortality of this population is very high. There was a high prevalence of co-infection in the CSF, indicative of severe immunodepression in this population. This finding warrant a more in dept study in the future since in resource-limited settings, patients present with advance immunodepression and the high morbidity and mortality associated with this condition will continue to bring great challenges to clinicians. Multiple pathogens infection in CNS disorder patients will be an important topic to assess with further study in larger cohorts and with subsequent validation of results.

Diagnostic testing for a broad array of CSF pathogens may be helpful in resource-limited settings to avoid unnecessary treatments and achieve better prognosis. For TB diagnosis, the Xpert MTD/RIF assay is a fully automated nucleic acid amplification testing (NAAT) that can deliver a result for MTB and rifampin resistance in about 2 h. On the basis of studies in countries with high tuberculosis burden, Xpert has been endorsed by the World Health Organization and widely deployed [21–28]. In contrast to other NAATs, Xpert can be performed on-demand by personnel with minimal training [28, 29]. The present study supports analysed suggesting that Xpert implementation in the United States is efficient and cost-effective [30]. Therefore, in resource-limited countries, establishment of Xpert is one of the ways to attempt improving the clinical diagnosis level about TB infection in CNS among AIDS patients.

Our study had some limitations. First, the generalizability of our research finding is limited. Since our hospital is the sole comprehensive hospital well-known for AIDS treatment in Hubei province, we can make some general assumptions regarding CNS infection in a province in China. Second, the number of specific pathogens for PCR testing was limited and there are other possible etiologies of CNS infection, but were not assessed in our study. Last but not least, the detection samples associated with the study were limited to CSF, which may be incomplete for diagnosis since pathogens can present in the CSF transiently. For many types of CNS infections, serologic detection on the presence of virus-specific IgM antibodies in serum or a rise in antibody titer can be useful; this study did not include serologic assays.

In this series, in addition to the molecular testing, Cryptococcus can be diagnosed with India Ink and CrAg, TB can be diagnosed with Xpert, and Toxoplasmosis can frequently be managed using an empirical strategy. Meanwhile, some pathogens such as EBV and JCV have no specific, effective treatment, while others such as CMV and HHV-6 require specialized therapies that are frequently unavailable in a resource limited setting. Disease burden, economic pressure, health resource and severity of diseases are critical factors which can effect the cost-efficacy of running a molecular laboratory. In China, we believed that the molecular testing can only be recommended under conditioned circumstances, rather than prioritized under all circumstances.

In conclusion, this study provides baseline data regarding the etiology distribution of CNS infections among hospitalized patients living with HIV/AIDS in a comprehensive Chinese hospital. It reported that the most etiology was cryptococcal meningitis (63.0%). Even as high as 22.2% of CMV DNA positive rate was found in this study, more than half were proved to be an innocent bystander. During periods of severe immunosuppression, AIDS patients with unrecognized CNS opportunistic infections are at high risk of death; thus, efforts should be focused in improving rates of pathogen diagnosis in this vulnerable group. CSF PCR, which is the standard diagnostic technique used in resource-rich settings to detect etiology infections of the CNS, is not currently used for suspected CNS disorder patients in China. Due to limited resources, whether CSF PCR should be prioritized needs to consider the patients’ economic ability.

## Abbreviations

CNS: central nervous system
AIDS: acquired immunodeficiency syndrome
CMV: cytomegalovirus
EBV: Epstein-Barr virus
JCV: JC polyomavirus
VZV: varicella-zoster virus
HHV-6: human herpesvirus 6
HSV: herpes simplex viruses
CSF: cerebrospinal fluid
